# Single-port laparoscopic Deloyers procedure for tension-free anastomosis after extended left colectomy or subtotal colectomy: A 6-patient case series

**DOI:** 10.1097/MD.0000000000021421

**Published:** 2020-07-31

**Authors:** Byung Jo Choi, Woojin Kwon, So Hye Baek, Won Jun Jeong, Sang Chul Lee

**Affiliations:** a Department of Surgery; b Department of Anesthesiology and Pain Medicine, College of Medicine, The Catholic University of Korea, Seoul, Korea.

**Keywords:** deloyers procedure, laparoscopy, right colon to rectal anastomosis, single-port

## Abstract

Right colon-to-rectal anastomosis is performed in relatively rare conditions, including after subtotal colectomy or extended left hemicolectomy. One technique of tension-free anastomosis is the Deloyers procedure that includes cranio-caudal rotation of the right colon. As with other colon surgeries, the laparoscopic approach has been adapted for the Deloyers procedure. Nevertheless, due to its rare indications and technical specificity, only a small case series have been reported. Here, we report our experience with single-port laparoscopic (SPL) Deloyers procedures.

Between June 2013 and March 2018, 6 patients underwent SPL Deloyers procedures. Three patients underwent SPL subtotal colectomy with ascending colon-to-rectal anastomosis for sigmoid colon cancer with chronic ischemic colitis, sigmoid colon cancer with left colon ischemia, and synchronous transverse and sigmoid colon cancer, respectively. The other 3 patients underwent SPL Hartmann reversal using the Deloyers procedure technique for 2 transverse colon end colostomies and 1 ascending colon end colostomy state, which were the result of a previous extended left hemicolectomy and subtotal colectomy, respectively. A commercially available single port was used with conventional straight and rigid laparoscopic instruments. The surgical procedures were similar to those performed during conventional laparoscopic surgery. For the anastomosis, the mobilized remaining ascending colon was rotated 180° counter-clockwise around the axis of the ileocolic pedicle. Tension-free colorectal anastomosis was then performed between the well-vascularized ascending colon and the rectal stump.

The SPL Deloyers procedure was successful in all patients. No additional incisions for trocars or conversions to open surgery were necessary. The operative time and postoperative length of stay were 210 to 470 min and 8 to 21 days, respectively. No intraoperative complications were noted. There were 3 minor postoperative complications without anastomotic leakage. All patients had 2 to 3 bowel movements per day, and 1 patient regularly took loperamide at 6 months after surgery.

The SPL Deloyers procedure was feasible and allowed patients to achieve good bowel movements. This operation may be considered an additional surgical option for experienced SPL surgeons in selected patients.

## 1 Introduction

The Deloyers procedure is a technique used as an alternative to total colectomy with ileorectal anastomosis after extended left-sided colonic resection. This technique consists of an anastomosis between the right or the transverse colon and the rectum or anus after complete mobilization and rotation of the right colon, preserving the ileocolic artery.^[1]^ Left-sided colonic resection extending to the transverse colon or rectum is rarely performed. However, in patients with synchronous colon cancer, metachronous colon cancer, ischemia of the left colon after ligation of the inferior mesenteric artery during colectomy, or synchronous pathology in the left colon and rectum, an extended resection or unexpected additional colectomy is inevitable. In these situations, when the residual colon cannot reach the rectal stump without tension, the Deloyers procedure can be used for tension-free anastomosis as a technique to avoid total colectomy and ileorectal anastomosis. Laparoscopic techniques have been employed for Deloyers procedure in an attempt to reduce morbidity and mortality.^[2,3]^ Nevertheless, to our knowledge, there are no reports on the use of single-port laparoscopic surgery (SPLS) for the Deloyers procedure. Therefore, we present the first 6 cases of SPL Deloyers procedure.

## 2 Methods

Between June 2013 and September 2018, 6 patients underwent the SPL Deloyers procedures at Daejeon St. Mary's Hospital, an affiliate of the Catholic University of Korea. All patients who were eligible for conventional laparoscopic procedures met the inclusion criteria. No patient was excluded from undergoing SPLS on the basis of the type and number of prior abdominal operations or body mass index. The indications for this technique were conditions requiring right colon-to-rectal anastomosis, followed by simultaneously or previously performed extended left hemicolectomy or subtotal colectomy. The exclusion criteria were severe medical illness and hemodynamic instability. All surgeries were performed by 1 surgeon who was well experienced in performing SPLS. The patients provided written informed consent prior to undergoing SPLS and for the publication of this paper. The study was approved by the institutional review board of Daejeon St. Mary's Hospital (IRB code: DC19RESI0091).

## 3 Patients

### 3.1 SPL Deloyers procedure for subtotal colectomy

Three patients underwent SPL subtotal colectomy with ascending colon-to-rectal anastomosis for sigmoid colon cancer with chronic ischemic colitis, sigmoid colon cancer with left colon ischemia, and synchronous transverse and sigmoid colon cancer, respectively. The first patient (Patient 1 in Tables 1–3) had descending colonic stricture as a sequela of chronic recurrent ischemic colitis, and the patient was diagnosed with sigmoid colon cancer on the follow-up colonoscopy study. The second patient (Patient 4) had undergone anterior resection for sigmoid colon cancer. However, during the operation, after inferior mesenteric artery ligation, left colon ischemic changes developed. For a secure anastomosis, the transverse colon was sacrificed and the Deloyers procedure was performed. The third patient (Patient 6) underwent the Deloyers procedure for double primary colon cancer.

**Table 1:**
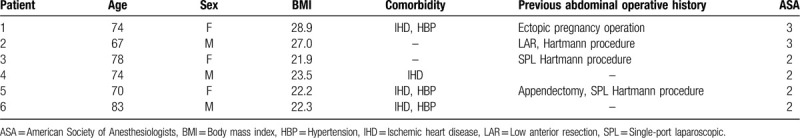
Demographics of patients undergoing single-port laparoscopic Deloyers procedure.

**Table 2:**
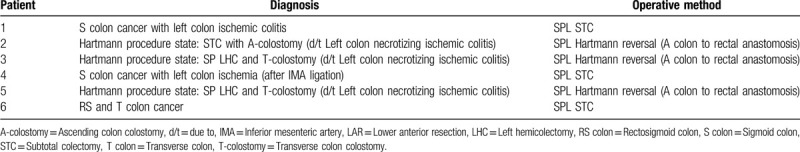
Operative methods details undergoing single-port laparoscopic Deloyers procedure.

**Table 3:**
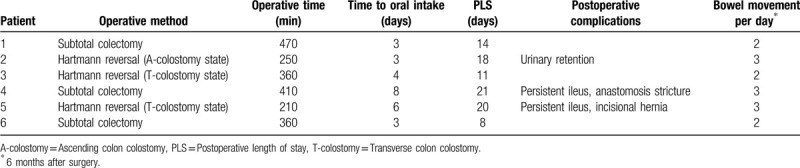
Operative and post-operative data of patients undergoing single-port laparoscopic Deloyers procedure.

### 3.2 SPL deloyers procedure as hartmann reversal

Another 3 patients underwent SPL Hartmann reversal using the Deloyers procedure technique for 2 transverse colon end colostomy (T-colostomy) and 1 ascending colon end colostomy (A-colostomy) state. The original Hartmann procedures were performed for left colon necrotizing ischemic colitis in all 3 patients. The first patient (Patient 2) underwent open subtotal colectomy, followed by A-colostomy for left colon necrotizing ischemic colitis, which was his original Hartmann procedure. He had a previous operative history of SPL low anterior resection (LAR) for upper rectal cancer at 8 months before the Hartmann procedure. The second and third patients (Patients 3 and 5, respectively) underwent extended left hemicolectomy as their original Hartmann procedures, followed by A-colostomy for left colon necrotizing ischemic colitis. These 2 patients with a transverse colon stoma had no previous surgery. In all 3 patients who had a stoma, Hartmann reversal using this right colon-to-rectal anastomosis techniques was performed to take down the colostomy, and they were included in this series for the evaluation of the feasibility and functional outcomes of this technique.

### 3.3 Surgical technique

**Figure 1:**
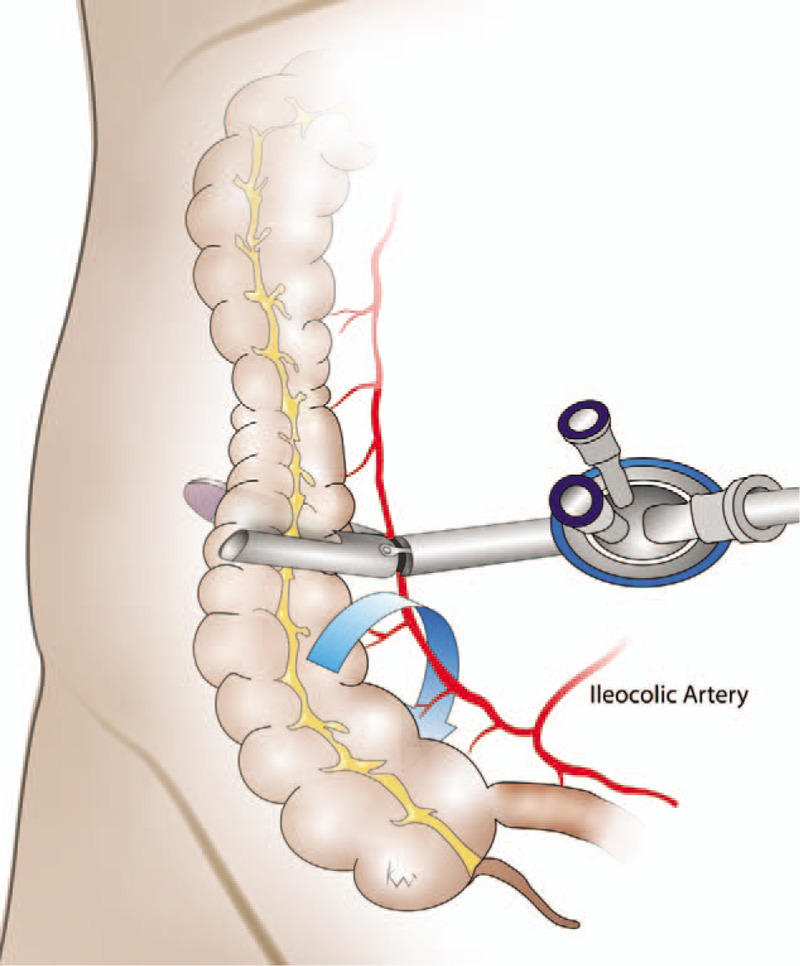
The surgical procedures with preservation of the ileocolic artery and transection of the right colon at the mid-portion level of ascending colon. The fully mobilized remaining right colon is rotated 180° counter-clock-wise around the axis of the ileocolic pedicle, so that its anterior aspect is flush on the retroperitoneum.

**Figure 2:**
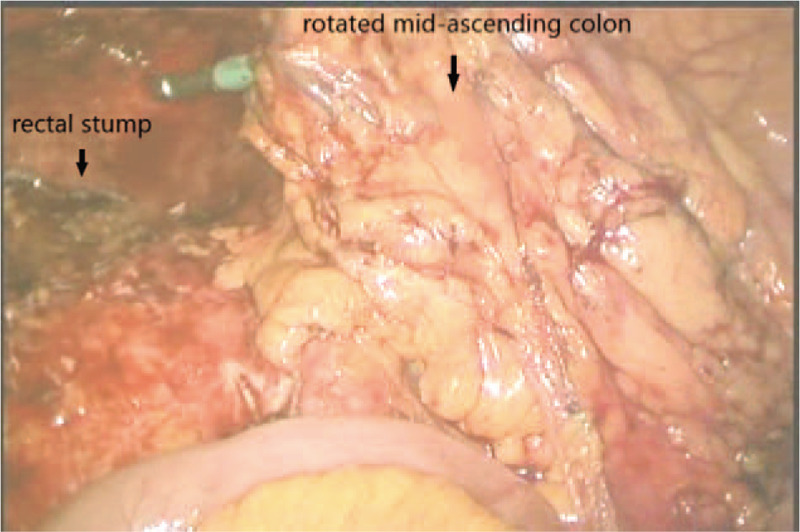
Laparoscopic view showing placement of the ascending colon after counter-clock wise rotation.

**Figure 3:**
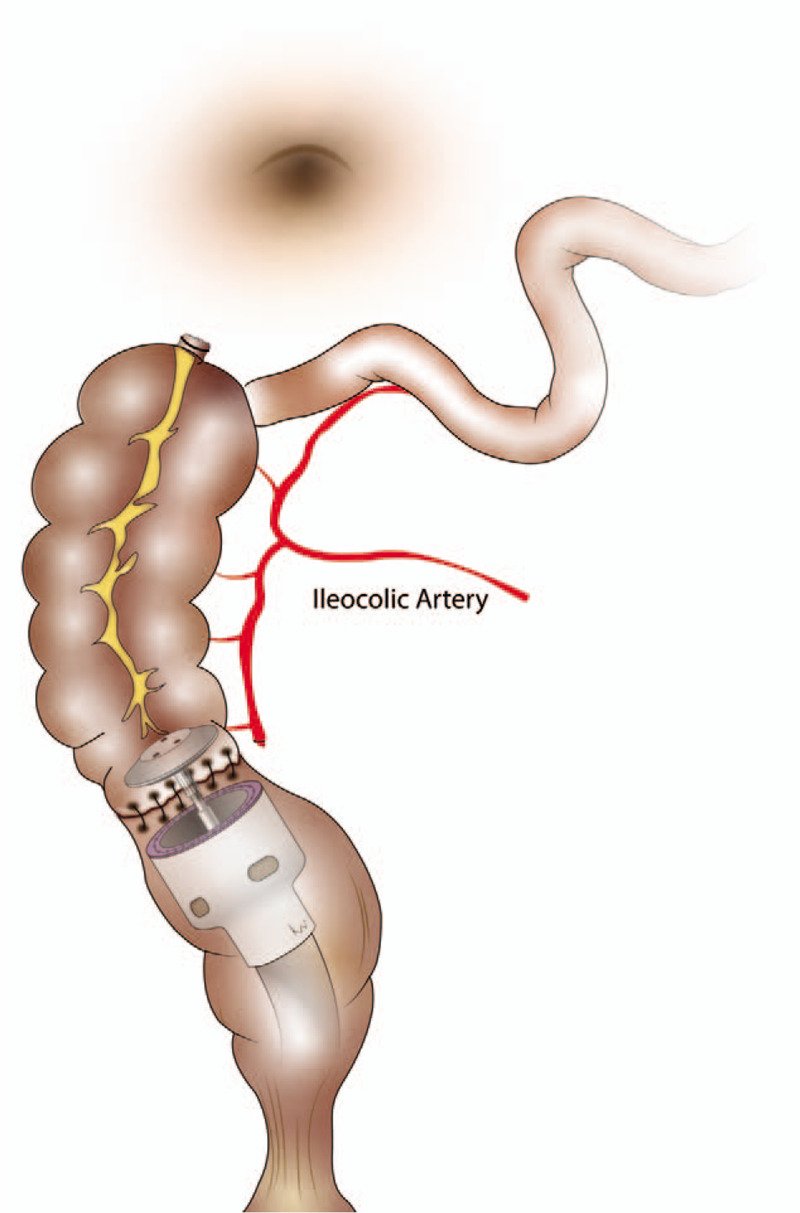
Tension-free colorectal anastomosis between the well-vascularized right colon and rectal stump in an end-to-end fashion.

The protocol for colon SPLS has been standardized, as reported previously in our institution.^[4,5]^ For SPL subtotal colectomy, the umbilicus was used as the single incision site, and a commercial single port (OCTO port; Darim, Korea) was utilized. The surgical procedures were similar to those performed during conventional laparoscopic surgery. The left colon was mobilized from the medial to the lateral side. After splenic flexure dissection, the transverse mesocolon was dissected, proceeding from left to right until the middle colic vascular pedicle was identified. Upon completion of the left colon mobilization, the operator moved to the left side of the patient and right colon dissection was conducted. The surgical procedures were also similar to those of conventional inferior-to-superior right colon dissection, except for the preservation of the ileocolic vascular pedicle (Fig. 1). High ligation of the inferior mesenteric artery and D3 lymph node dissection involving ligation at the root of the middle colic artery was routinely performed in patients with cancers of the sigmoid colon or transverse colon. After full colon mobilization including the distal ileum, transection of the right colon was performed and the specimen was removed via the single incision site. For anastomosis, the fully mobilized remaining right colon was rotated 180° counter-clock-wise around the axis of the ileocolic pedicle (Fig. 2). Tension-free colorectal anastomosis was then performed between the well-vascularized right colon and rectal stump in an end-to-end fashion (Fig. 3).

In SPL Hartmann reversal using this technique, the stoma site was used as the single-incision site. Two of the 3 Hartmann reversal cases had a T-colostomy stoma on the umbilicus after a prior SPL extended left hemicolectomy, and 1 had an A-colostomy stoma on the right lower quadrant after a previous open subtotal colectomy. The stoma was first mobilized down to the fascia and completely detached from the abdominal wall. After direct-vision adhesiolysis of peritoneal adhesions around the stoma site, we inserted a commercial single port. In all 3 cases, ascending colon-to-rectal anastomosis was performed. In 2 cases with a T-colostomy stoma, right colon mobilization was required. After complete right colon and distal ileum mobilization, transection of the transverse colon stump and right colon at the mid-portion level of the ascending colon was performed, and the specimen was removed via the single incision site. The proximal colon stump was extracorporealized through the single-incision site, and an optimally-sized anvil of a circular stapler was secured in the proximal colon. The prepared colon was then returned to the peritoneal cavity. Then, anastomosis between the ascending colon and the remnant rectum was performed using the technique described above.

## 4 Results

The SPL Deloyers procedure was successful in all patients. No additional incisions for trocars or conversions to open surgery were necessary. None of the patients required a temporary loop ileostomy. Patients’ demographics and the indications for the Hartmann's procedure are shown in Table 1. Four patients had ischemic heart disease as a comorbidity. In the Hartmann reversal group, prior Hartman's procedure was performed by SPLS in 2 patients and by the open method in 1. The operative details and operative and postoperative outcomes are shown in Tables 2 and 3. All patients had 2 to 3 bowel movements per day, and 1 patient regularly took loperamide at 6 months after surgery.

No intra-operative complications were noted. Three minor postoperative complications were observed, which are as follows: postoperative ileus (n = 2) and urinary retention (n = 1). These complications resolved with conservative management. Postoperative anastomotic site leakage was not observed. There were 2 cases of re-operations for late complications. One patient (Patient 4) had an anastomotic stricture at 11 months after the SPL Deloyers procedure that required transanal dilatation. At 22 months after the transanal dilatation, the stricture reoccurred. The patient underwent stricture resection and re-anastomosis, which was performed by SPLS, and he was well upon discharge at 7 days after the reoperation. The other patient (Patient 5) who underwent SPL Hartmann reversal via an umbilical single-incision developed an incisional hernia at the single incision site, which was treated with single-port herniorrhaphy and dual phase on-lay mesh at 7 months after the Deloyers procedure. Coincidentally, these 2 patients both experienced ileus as early postoperative complications.

The median follow-up duration was 39 months (range: 10–74). During the follow-up period, 1 patient (Patient 5) was lost to follow-up at 14 months after the operation and 1 (Patient 4) patient died due to cancer recurrence and aggravation at 29 months after the operation. The remaining patients were well at their latest follow-up visits.

## 5 Discussion

The present case series showed that the use of SPLS for the Deloyers procedure is a safe and feasible technique associated with low morbidity and acceptable bowel function.

Despite the fact that the Deloyers procedure was first developed to treat ulcerative colitis, megacolon, dolichocolon with chronic constipation, and polyposis involving the left and transverse colon by Deloyers in 1963,^[6]^ the procedure is used today for a variety of other indications, including synchronous and metachronous colon cancers. Since the right colon, ileocecal valve, and distal ileal loop are preserved, the Deloyers procedure can achieve better functional outcomes than the ileorectal anastomosis. Previous reports, including a large-scale analysis of 48 patients using the open approach, demonstrated good functional results, with fewer than 4 bowel movements per day in all patients.^[2,7,8]^ The remaining physiological reservoir function permits sustaining the metabolism of undigested starch and the production of short-chain fatty acids, resulting in the normal absorption of sodium and vitamin B12 as well as prevention of renal and gallbladder lithiasis.^[9,10]^ In terms of postoperative complications, the Deloyers procedure appears to be superior to total colectomy with ileorectal anastomosis. Two large-scale studies of the Deloyers procedure, involving 48 and 14 patients, respectively, reported no leakage.^[7,8]^ A study of the laparoscopic approach including 10 patients reported only 1 patient with anastomotic leakage.^[2]^

Since the first single-port colectomy was performed in our institution in March 2009, SPLS for colorectal disease including malignancies has been routinely conducted as a minimally invasive procedure. In the minimally invasive surgery center at our institution, the SPL Deloyers procedure is performed for patients with conditions requiring extended left hemicolectomy, as it is minimally invasive and has good postoperative outcomes. In the present case series, we obtained acceptable results in 6 patients who underwent the SPL Deloyers procedure for various conditions. There were no major complications, including anastomotic leakage, and all patients had good functional outcomes with less than 3 bowel movements a day and solid stool consistency. However, the complication rate (including minor events) was relatively high (50%).

There are limitations to our case series. In all patients, the right colon-to-rectal anastomosis was performed. There were no colo-anal anastomoses. The second patient had a history of LAR; nevertheless, we were able to anastomose with the distal rectum. This might have a positive effect not only on the functional outcomes, but also on the postoperative outcomes. In addition, we performed resection and anastomosis in the mid-ascending colon level when we resected the right colon for anastomosis. This was done because we believe that the rotation of the relatively short length of the colon segment was technically easier and that it would provide adequate blood supply. This technique resulted in good bowel movements at 6 months after surgery. However, we only assessed the bowel movement after the operation. The absence of a single tool measuring the various functional outcomes is another limitation. When the transverse colon remains after extended left hemicolectomy, transmesenteric lowering, consisting of taking down the proximal colon through a jejunal avascular mesenteric window, may be an alternative to the Deloyers procedure.^[11]^ Another alternative for colonic salvage (i.e., antiperistaltic cecorectal anastomosis) has been reported.^[12]^ Nevertheless, these have drawbacks, such as the risk of internal herniation through the mesenteric window and dual anastomosis and loss of ileocecal valve, respectively. Therefore, we prefer the Deloyers procedure, despite creating torsion of the ileocolic pedicle during colonic transposition. In the present cases, complete mobilization and cranio-caudal rotation of the right colon did not result in any torsion of the vascular pedicle, as reported in previous studies.

In our series, the operative times for 3 SPL subtotal colectomy cases using the Deloyers technique were similar to the median operative time of 415 minutes reported by Manceau et al,^[7]^ but it was longer than the median operative times of 228 and 189 minutes reported by Kontovounisios et al^[8]^ and Sciuto et al^[2]^, respectively; all of their cases underwent laparoscopic procedures. Nevertheless, this is the initial single-port case series of the Deloyers procedure. Confirmatory results may be obtained through larger cases series with careful selection of patients.

We previously reported the safety and efficacy of the SPL Hartmann reversal in patients with left colon end-colostomy.^[13]^ In the current case series, 3 cases of SPL Hartmann reversal using the Deloyers technique are included, and we demonstrated a successful alternative surgical option, SPL Hartman reversal using Deloyers technique, in a patient with left colon end colostomy, which is also suitable for patients with rare conditions that require right colon and rectum anastomosis. Of course, it should be considered that the original Hartmann procedure was implemented using single-port laparoscopic techniques in 2 of these 3 SPL Hartmann reversal cases.

Moreover, it is noteworthy that 2 of the 6 patients had left colon ischemia after inferior mesenteric artery ligation and underwent a subtotal colectomy. One case of left colon ischemia occurred at 8 months after LAR, whereas the other case was identified shortly after inferior mesenteric artery ligation during surgery. One patient had no vascular disease as comorbidity. The evaluation of collaterals should be carried out perioperatively, including preoperative vascular evaluation using imaging studies and intraoperative direct collateral identification or indocyanine green-enhanced fluorescence tests.

In conclusion, the Deloyers procedure has few surgical indications. Nevertheless, if necessary, it could be performed as a single-port laparoscopic procedure, without causing additional complications. The SPL Deloyers procedure allows patients to achieve good bowel movements, and it may be considered an additional surgical option for experienced SPLS surgeons in selected patients. Nevertheless, our findings warrant further investigation using larger cohorts.

## Author contributions

**Conceptualization:** Sang Chul Lee.

**Data curation:** Byung Jo Choi, So Hye Baek.

**Formal analysis:** Byung Jo Choi.

**Methodology:** Sang Chul Lee.

**Resources:** Won Jun Jeong.

**Software:** Woojin Kwon.

**Visualization:** Woojin Kwon.

**Writing – original draft:** Byung Jo Choi.
